# Dynamic Stability of Volatile Organic Compounds in Respiratory Air in Schizophrenic Patients and Its Potential Predicting Efficacy of TAAR Agonists

**DOI:** 10.3390/molecules28114385

**Published:** 2023-05-27

**Authors:** Anna Held, Dariush Henning, Carina Jiang, Christoph Hoeschen, Thomas Frodl

**Affiliations:** 1 Department of Psychiatry, Psychotherapy and Psychosomatics, University Hospital, RWTH Aachen, 52074 Aachen, Germany; 2 Department of Psychiatry and Psychotherapy, Otto von Guericke University Magdeburg (OVGU), 39106 Magdeburg, Germany; 3 Institute of Medical Engineering, Otto von Guericke University Magdeburg (OVGU), 39106 Magdeburg, Germany

**Keywords:** volatile organic compounds, schizophrenia, breath-analysis

## Abstract

Objectives: Volatile organic compounds (VOCs) in the breathing air were found to be altered in schizophrenia patients compared to healthy participants. The aim of this study was to confirm these findings and to examine for the first time whether these VOCs are stable or change in concentration during the early treatment course. Moreover, it was investigated whether there is a correlation of the VOCs with the existing psychopathology of schizophrenia patients, i.e., whether the concentration of masses detected in the breath gas changes when the psychopathology of the participants changes. Methods: The breath of a total of 22 patients with schizophrenia disorder was examined regarding the concentration of VOCs using proton transfer reaction mass spectrometry. The measurements were carried out at baseline and after two weeks at three different time points, the first time immediately after waking up in the morning, after 30 min, and then after 60 min. Furthermore, 22 healthy participants were investigated once as a control group. Results: Using bootstrap mixed model analyses, significant concentration differences were found between schizophrenia patients and healthy control participants (*m*/*z* 19, 33, 42, 59, 60, 69, 74, 89, and 93). Moreover, concentration differences were detected between the sexes for masses *m*/*z* 42, 45, 57, 69, and 91. Mass *m*/*z* 67 and 95 showed significant temporal changes with decreasing concentration during awakening. Significant temporal change over two weeks of treatment could not be detected for any mass. Masses *m*/*z* 61, 71, 73, and 79 showed a significant relationship to the respective olanzapine equivalents. The length of hospital stay showed no significant relationship to the masses studied. Conclusion: Breath gas analysis is an easy-to-use method to detect differences in VOCs in the breath of schizophrenia patients with high temporal stability. *m*/*z* 60 corresponding to trimethylamine might be of potential interest because of its natural affinity to TAAR receptors, currently a novel therapeutic target under investigation. Overall, breath signatures seemed to stable over time in patients with schizophrenia. In the future, the development of a biomarker could potentially have an impact on the early detection of the disease, treatment, and, thus, patient outcome.

## 1. Introduction

Schizophrenia is a mental illness characterized by a variety of different symptoms and associated with high levels of functional impairment in all areas of life [1]. Lifetime prevalence worldwide was reported to be 1% for decades, with onset most commonly reported between the ages of 15 and 35, with women developing the disease later than men on average [2]. Recent studies were able to somewhat specify the epidemiological data. For example, these studies showed an incidence of 15 in males and 10 in females per 100,000 population, a mean lifetime prevalence of 4 per 1000 population, and a lifetime morbidity of 0.7% [3]. The causes of schizophrenia are not fully understood but appear to be multifactorial. For example, genetic, environmental, and neurobiological factors play a role in the development of schizophrenia [1,4]. Clinically, the differentiation between major psychoses such as schizophrenia and other psychiatric disorders such as schizoaffective disorder or bipolar disorder can be challenging, especially when patients with these present psychotic features. Moreover, during the early stages of disease onset, the correct diagnosis sometimes only becomes evident with the ongoing course of the disease. Currently, the diagnosis of major psychiatric disorders is based on psychopathology and history according to Diagnostic and Statistical Manual of Mental Disorders (DSM-5) criteria and may lack neurobiological and, thus, mechanistic validity. It is, therefore, not surprising that research to find valid biomarkers for psychiatric disorders was found to be steadily increasing.

For example, a large number of studies were conducted in the past to investigate metabolic biomarkers. These were summarized in a systematic review by Davison from 2018 [3], which was dedicated, in particular, to the field of metabolomics. Accordingly, striking differences in essential polyunsaturated fatty acids, particularly arachidonic acid, were found. These were found to be lower in schizophrenia patients compared to healthy subjects. Cortisol was also the subject of many studies and significant differences were found in the waking response between healthy and schizophrenia patients. Accordingly, schizophrenia patients had a greater increase in cortisol during waking than healthy control participants. Vitamin E and creatinine, on the other hand, were found to be decreased in schizophrenia patients compared to healthy subjects in various studies. Cytokines in the context of inflammatory processes were also investigated. According to this, significantly higher levels of interferon-alpha, IL-1RA, IL-1ß, IL-6, IL-8, IL-12, TNF-alpha, and sIL-2R, were detected in the blood of patients acutely ill with schizophrenia compared with healthy control subjects [5]. A 2018 review by Orsolini et al. summarized a variety of studies that examined the role of inflammatory processes and immune dysfunction represented in C-reactive protein in schizophrenia patients. Accordingly, significantly elevated levels of C-reactive protein were shown in schizophrenic patients [6]. In addition, the elevated levels also correlated with the severity of symptoms [6]. In a meta-analysis by Kambeitz et al., 2015, a total of 1602 MRI scans of participants with first episode or chronic schizophrenia were studied to develop a biomarker based on neuroimaging. The results showed with a sensitivity and specificity of both 80% functional and structural changes in the brain of schizophrenic patients compared to healthy participants [7]. Moreover, the brain-derived neurotrophic factor (BDNF) was described as a potential blood-based biomarker regarding diagnosis as well as treatment of schizophrenia [8].

However, to date, no potential biomarker was integrated into clinical practice despite the large number of studies. None of these biomarkers were tested in clinical approval trials yet, they were not tested in different patient samples, and sensitivity and specificity seem to be too small of these biomarkers to be used in clinical practice. So far, no biomarker was fully convincing in terms of generalisability, replicability, or clinical applicability and utility. A valid biomarker is characterized by reliability, plausibility, accuracy, and reproducibility [9]. In particular for schizophrenia, the specificity needs to be very high so that not many participants without schizophrenia receive a suspicious diagnosis of schizophrenia.

It might be possible to establish so-called volatile organic compounds (VOCs) in the breath as a possible biomarker that could provide information about altered metabolic pathways related to the disorder or side effects of therapy and, thus, might provide valid information for the clinician. Metabolomics recently emerged as a particularly potential quantitative tool that provides specific multi-parametric biomarker signatures reflecting alteration in an array of biochemical processes that underpin psychiatric disorders [10] and the effects of drug therapies on those biological processes [11]. It is the end point of the omics cascade and reflects the products from the alterations that occur at the genetic, transcriptional, and translational levels, as well as perturbation in gut microbiome metabolic and global metabolic response of environmental influence. Breathomics is one of the newest branches of metabolomics to explore the metabolic change that are related to disorders. Compared to other biomatrices-based metabolomics, breathomics offer practical advantages. Breath samples are suitable for frequent and, thus, longitudinal monitoring of biomarkers related to disease progression, treatment response or side effects and can be assessed in real time and analyzed in a matter of minutes making breath gas analysis rapid and cost-effective. The investigated VOCs in the breath could also underline the close interactional connection between the brain and the lungs (in the sense of the so-called “organic-crosstalk”). For example, breath can provide information about the intestinal flora, and about this, in turn, it can provide information about the diet of the participants. It could, for example, be suspected that isoprene, as an important precursor in cholesterol biosynthesis, could show stability in the breath analyses [12].

Breath gas analysis in schizophrenia was already the subject of a few studies. Philips et al. studied 25 schizophrenia patients, 26 patients with other psychiatric disorders, and 38 healthy control participants by breath analysis using gas chromatography combined with mass spectroscopy (GC-MS). Among others, markers such as 2-methylbutanes, trichlorofluoromethanes, 2-pentanol, pentanes, dichloromethanes, trichloroethenes, benzenes, I-chloro-2-methylbutanes, 2,3,3-trimethylpentanes, 2,2-dimethylbutanes, and tetrachloroethenes were studied. There were significant differences in VOCs in the breath of schizophrenia patients compared to the two control groups [13]. Ross et al. specifically examined alkanes, ethane, and butane in the breath of schizophrenia patients compared to healthy control participants and showed that concentrations of ethane and butane were elevated in schizophrenia patients [14]. The analysis was restricted to just these two VOCs and the group of patients was mixed from patients with schizophrenia and bipolar disorder. Popa et al. investigated a limited number of VOCs in 15 schizophrenia patients and 19 healthy control participants. For this purpose, the ethylene and ammonia concentrations from breath samples before/after treatment with levomepromazine of the schizophrenia patients were measured and compared with the breath samples of the healthy control participants. The ethylene and ammonia concentrations were found to be significantly increased in the schizophrenia patients compared to the healthy control group [15].

By Jiang et al., in the same study on the cross-sectional baseline data, the concentration of VOCs by breath analysis in schizophrenia patients was compared with a healthy control group and it was found that trimethylamine (*m*/*z*) was most significantly altered in concentration among nine other masses (*m*/*z* 39, 40, 59, 69, 70, 74, 85, 88, 90) in schizophrenia patients compared to healthy control participants. No significant differences were found with respect to gender and BMI. Similarly, no significant association was found with olanzapine equivalents, duration of illness, and length of hospital stay. Based on three masses (60, 85, and 90), a good classification of the diagnostic groups in the sample was found, but it must be added restrictively that the number of participants was too small to generalise the results. Nevertheless, analysis of breath using proton transfer reaction mass spectrometry looks promising to help developing a biomarker. The examination itself has many advantages. It works simply, so patients can usually even perform the breath sampling on their own. It is fast, non-invasive, and can be performed frequently [16]. The changes in trimethylamine seem to be an interesting approach for the development of possible biomarkers but also new psychotropic drugs. Our studies aim to confirm and further substantiate these changes. These previous studies had no longitudinal measurements and, thus, were not able to show VOC-stability over a 4-week period, which is important in terms of reliability and reproducibility when developing a biomarker.

In this study, breath gas analyses were performed in schizophrenia patients with a longitudinal design over 14 days using proton transfer reaction mass spectrometry (PTR-MS) and analysed for VOCs. The Proton transfer reaction mass spectrometry is an analytical method that was invented and developed in 1995 at the Institute of Ion Physics at the Leopold Franzens University in Innsbruck, Austria. It is an analytical chemistry technique that can detect and measure volatile organic compound in ambient or breath air. Here, hydronium reagents are used in the gas phase to generate ion sources. The ion source is connected to a drift tube and to an analytical system. H_3_O^+^ ions are used as the reagent. Proton transfer will happen from H_3_O^+^ ions to all components with a higher proton affinity than water [17]. The PTR-MS is a highly sensitive analytical method for the detection of VOCs and can analyse concentration changes in small time intervals [18].

In 2020, the peppermint initiative sought to establish comparability of breath sampling and analysis methods and to increase the reliability of such tests. This is to be based on a common approach to monitoring a controlled intervention that takes into account the inherent biological variability of all breath data. It aims to establish benchmark values that could lead to finding out more about reproducibility and sensitivity of the analytical method. By determining washout profiles of different substances, e.g., menthone, as well as frequency and feature intensity distributions, an overview of the performance of a technique for breath analysis can be determined. The study aimed to provoke a transient and well-characterized disturbance in the VOC respiratory profile of participants by ingesting peppermint oil capsules. In the results, the kinetics of the leaching profiles of the participants showed similarities. The differences in absolute values could be due to differences in metabolism, e.g., BMI, age, diet. For menthone, for example, a mean washout time of 39.3 (±18.6) was determined [19].

In our study, we investigated the difference in breathing air between schizophrenia patients and the temporal dependence during the awakening period and within 14 days. The aim of the study was firstly to find out whether the so-called VOCs in the breathing air differ between schizophrenia patients and healthy controls and whether those of schizophrenia patients are stable or change in concentration during the waking period whether the compounds remain stable after two weeks. Moreover, it was investigated whether there is a correlation of the VOCs with the existing psychopathology of schizophrenia patients, i.e., whether the concentration of the masses detected in the breath gas alters when the psychopathology of the participants changes. Regarding the test quality criteria, the study of test–retest reliability was also calculated, in which it was examined whether the results of the tests are reproducible. The current study could also provide an indication about the disease process of schizophrenia.

## 2. Results and Discussion

### 2.1. Results

As shown in Table 1, there were 13 male participants and 9 female patients participating in the measurements at both time points. Regarding the body mass index (mean BMI 32.87 kg/m^2^, standard deviation 3.71 kg/m^2^) and age (minimum age 19 years, maximum age 53 years, standard deviation of 9.762 years), there were no changes due to the short time interval of two weeks between the two measurements. The distribution of the medication group and the division into smokers and non-smokers (13 smokers vs. 9 non-smokers) also showed no change.

The psychopathology of the patients was assessed using the PANSS score and the BDI-II score at both measurement time points. The dependent T-test showed significant improvements in the PANSS total (t = 2.371, *p* = 0.014, d = 2.452), PANSS positive (t = 2.881, *p* = 0.005, d =1.164), and the BDI-II (t = 3.930, *p* = 0.0004, d = 4.612) from the first measurement time point to the second measurement time point. Thus, both total psychotic and positive psychotic symptomatology and comorbid existing depressive symptomatology showed significant improvement in patients within two weeks.

The PANSS negative scores did not change significantly.

Mixed-model analysis was used to investigate whether there were VOC concentration differences between schizophrenia patients and healthy control participants with regard to the various masses in the breath. As shown in Table 2, the masses *m*/*z* 19, 33, 59, 60, 69, 74, and 89 showed significantly lower concentrations in the breath of schizophrenic participants compared to healthy participants (see Table 2). In contrast, the concentration of mass *m*/*z* 42 and 93 were significantly increased in schizophrenia patients. Even after the exclusion of the smoking participants, the result for the masses *m*/*z* 33, 60, 69, 74, and 89 remained significant and stable.

The test–retest reliability was significant for the masses *m*/*z* 19, 33, 35, 42, 59, 60, 63, 69, and 79. Thus, a stable reproduction of the results at the second measurement time point was possible for these masses (Table 3). Moreover, we provided Pearson correlations between the masses in the Supplemental Materials in order to see the common variance between them (Supplementary Table S2).

Using bootstrap mixture model analyses, the temporal change of the VOCs was additionally analysed during wake-up, after 30 min, after 60 min, and again at the mentioned three time points after two weeks.

Significant time effects (temporal change) over two weeks could not be detected for any mass.

**Figure 1:**
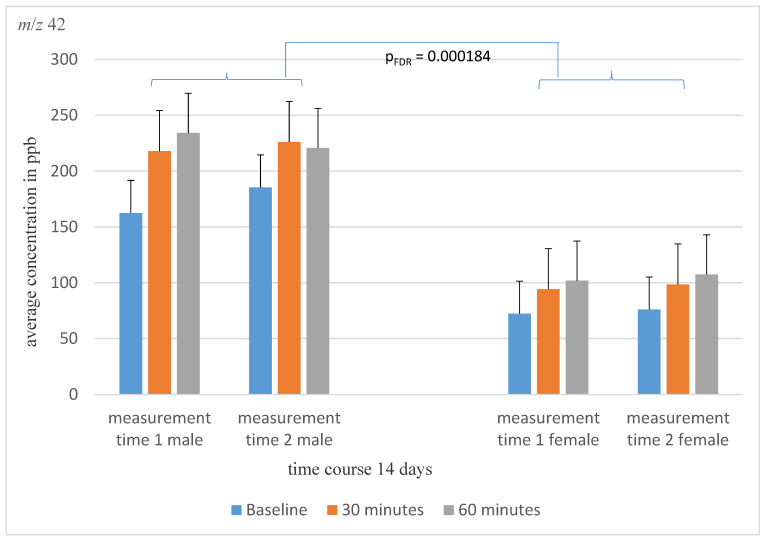
The average concentration of mass *m*/*z* 42 of male and female schizophrenia patients at the three different measurement time points over a 14-day time course. There is a significant concentration differences between the sexes.

However, a significant effect was found for differences between the sexes. Accordingly, possible differences in respiratory gas analysis between the sexes were investigated. Significant concentration differences between the sexes could be found for *m*/*z* 42 (Figure 1; *m*/*z* 42: p_FDR_ = 0.000184), *m*/*z* 45 (p_FDR_ = 0.0322), *m*/*z* 57 p_FDR_ = 0.02875), *m*/*z* 69 (p_FDR_ = 0.023), and *m*/*z* 91 (p_FDR_ = 0.02875).

**Figure 2:**
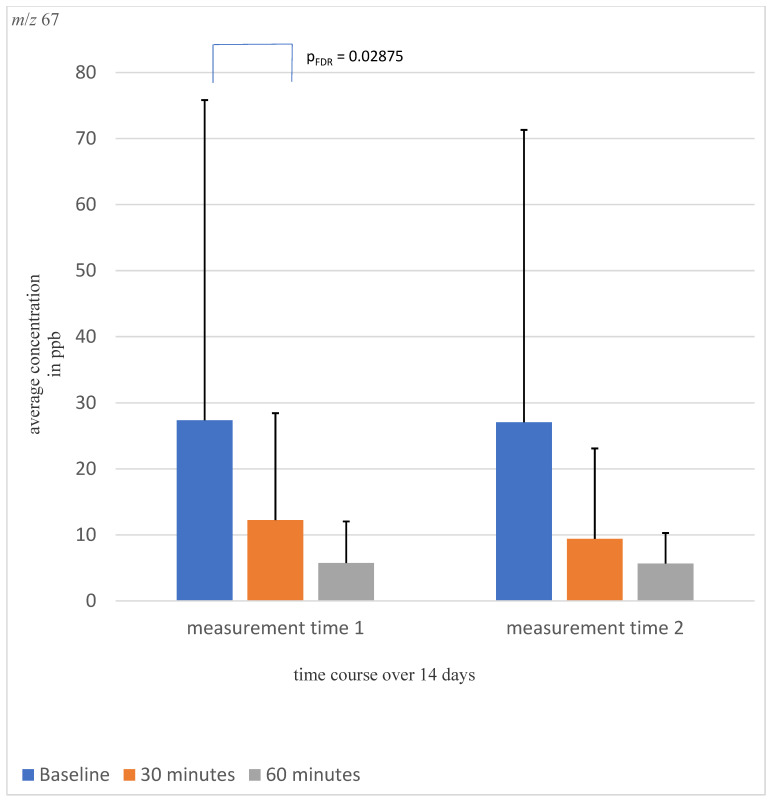
Shown is the average concentration of mass *m*/*z* 67 at the three different measurement time points over a 14-day time course. A significant awakening effect was detected.

A significant awakening effect was detected for concentrations of *m*/*z* 67 (p_FDR_ = 0.02875, shown in Figure 2) and *m*/*z* 95 (p_FDR_ = 0.02875, shown in Supplementary Figure S1). The other tested VOCs did not show significant time, awakening, or time by awakening effects.

**Figure 3:**
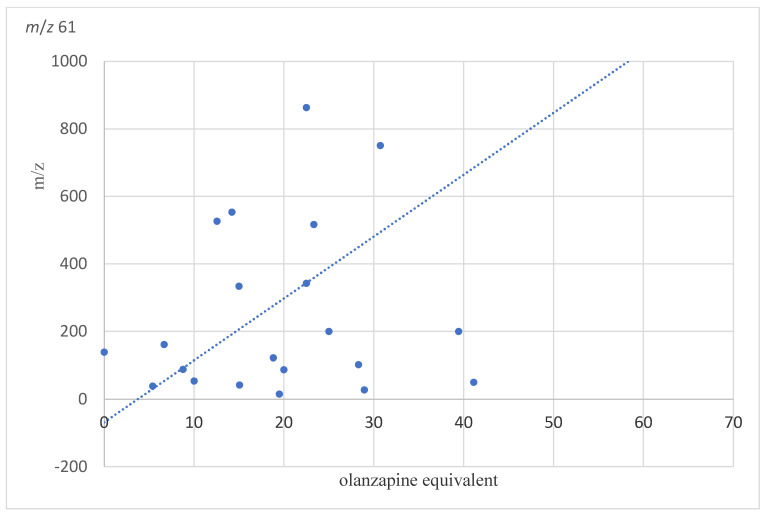
Shown is the Pearson correlation between the average concentration of mass *m*/*z* 61 and the olanzapine equivalents.

For masses *m*/*z* 61 shown in Figure 3 (r = 0.59, p_FDR_ = 0.032), *m*/*z* 71 (r = 0.68, p_FDR_ 0.0126, Supplementary Figure S2), *m*/*z* 73 (r = 0.63, p_FDR_ = 0.024, Supplementary Figure S3), and *m*/*z* 79 (r = 0.57, p_FDR_ = 0.036, Supplementary Figure S4), a significant correlation was detected to the respective olanzapine equivalents. The other masses did not show significant associations with olanzapine equivalents. In contrast, the length of hospital stay showed no significant relationship to the masses studied.

### 2.2. Discussion

So far, there are no biomarkers that simplify a diagnosis or support a decision about the prognosis of a schizophrenia disease. Recently, we could identify some VOCs that might have the potential to provide information about schizophrenia and help in the diagnostic and therapeutic purpose [16]. For such VOCs, it is highly important that they are relatively stable with regard to changes during the day and over several days during the therapy and, thus, that they are replicable. Therefore, the present study adds to the literature, showing that VOCs relevant for schizophrenia are stable over time, show a good test–retest reliability, and are valid with regard to associations to symptoms of the disease. Thus, breath gas analysis appears to be a simple, in large patient collective, feasible, and comparatively inexpensive method to detect changes in volatile organic particles.

The fact of the missing biomarker as well as the volatile organic compounds are already the subject of numerous research projects and studies. In a work of M. Philips, volatile organic compounds in the alveolar respiratory air of schizophrenia patients (N = 25) were examined and compared with other psychiatric patients (N = 26) as well as with healthy control participants (N = 37) [13]. At this time, the analysis was limited to a few substances that could be measured. In the results, there were no significant differences between the three groups in age, sex, tobacco use, or ethnicity. There was also no significant difference between the two patient groups with psychiatric history regarding the use of neuroleptics. However, there were significant differences in the volatile substances in the breath of schizophrenia patients compared to the two control groups. Thus, analysis of VOCs in breath in combination with pattern recognition analysis demonstrated identification of schizophrenia patients with a sensitivity of 80% and a specificity of 61.9%. For example, the substance pentane was detected in higher concentrations in the breath of schizophrenia patients, possibly as a sign of accumulation of oxygen free radicals that accelerate peroxidation of membrane lipids [13]. In the previous study by Jiang et al., 2022, the AUC of 0.96 and an accuracy of 91% was found to be higher based on data from three different masses [16].

In a previous study, we found trimethylamine (TMA) (*m*/*z* 60) to be significantly decreased in patients with schizophrenia [16,20]. Here, we were able to confirm this finding and we found that TMA was stable over the 14-day period and over several measurement times during the awakening period. Test–retest reliability was high and, thus, based on these investigations, TMA is a potential stable trait signature for schizophrenia. This is even more important since trace amines such as TMA are currently the subject of intensive research [21]. These endogenous chemical messengers are closely related to neurotransmitters such as dopamine, serotonin, and norepinephrine and have low concentrations in the central nervous system. In the development of new antipsychotic drugs, selective TAAR1 agonists showed promising results so far. They showed inhibitory effects on dopaminergic and serotonergic neuronal activity, but motor or metabolic side effects were less pronounced compared with previously used antipsychotics. TAAR-agonists also proved effective in treating negative symptomatology and cognitive symptoms in schizophrenia patients [21]. It should be noted here that the mass *m*/*z* 60 is normally also an isotope of *m*/*z* 59. The correlation of the two masses showed a value of 0.784, which is, in principle, very high. However, the correlation alone does not allow a statement about causality. This point needs further investigation, which will be included in subsequent studies already in progress.

A four-week randomized controlled trial by Koblan, Kent et al. compared the treatment of patients with an acute exacerbation of schizophrenia with a drug with a mechanism of action that did not bind to D2 receptors but had agonistic activity at TAAR1 and 5-HT1A receptors or with placebo. The results showed that patients treated with a TAAR1 and 5-HT1A receptor agonist had a greater reduction in PANSS total score than patients treated with placebo [22]. This shows that the analysis and identification of VOCs can also play a role in the development of new pharmacological therapy. Some masses showed a significant difference in our calculations with respect to their concentration in the breath of schizophrenia patients compared to healthy control participants. Even after the exclusion of smokers, the results for the masses *m*/*z* 33, 69, and 89 remained stable, so that a possible influence of smoking on the breath analysis could be excluded.

For example, by identifying the different masses (e.g., TMA = *m*/*z* 60) and measuring the concentration in the breath of patients, a biomarker could be developed for early detection of, for example, schizophrenia, but also as a therapy monitoring of psychopharmacological therapy in patients. Importantly, VOCs in breath were found to be temporal stable over 14 days and also during improvement of symptoms, so that they might be related to a trait of the disease rather than to a certain disease state. In further studies, it whether patients with schizophrenia and decreased TMA concentrations might respond better to TAAR receptor agonists must be tested.

In our calculations, the mass *m*/*z* 19 also showed significantly lower values in schizophrenia patients than in the healthy participants. *m*/*z* 19 is protonated water (H_3_O+), which cannot be counted as VOCs. The reasons for the differences shown can be speculated. Among other things, it is conceivable that the humidity of the exhaled air could play a role and that schizophrenia patients under antipsychotic medication with the associated anticholinergic side effects suffer from dry mouth and, therefore, their exhaled air was drier than that of healthy participants. In addition, the long hospital stay of the patients during the breath measurements might be another influencing factor.

This study was not without limitations. The role of microbiotic signalling was a recurring theme in this study and was the target of many previous studies, yet most of its signalling pathways, signal transduction, and overall function remain to be deciphered and understood. An important factor that influences the composition of a human’s microbiome is diet. Since TMA (*m*/*z* 60), butyric acid (*m*/*z* 90), and N-butylamine (*m*/*z* 74) are derived from dietary sources through the microbiome, a stable breath gas concentration found in the present study showed stability during a hospital stay with the diet not changing. No significant association was found which pointed to a more complex pathogenesis.

Using shotgun metagenome sequencing, a 2015 study by Castro-Nallar analyzed the oropharyngeal microbiome between schizophrenia patients and healthy controls. For example, it showed an increase in lactic acid bacteria (including Lactobacillus, Bifidobacterium), Candia, and Eubacterium in schizophrenia patients compared to healthy control participants. Neisseria, Haemophilus and Capnoytophaga, on the other hand, were found to be decreased, although it should be noted that a connection with smoking could not be excluded [23]. The analysis of the oropharyngeal microbiome could, therefore, also be used to draw conclusions about the intestinal microbiome. An influence on the respiratory gas analysis also seems likely and should be included in subsequent investigations of the VOCs.

Medication may have also influenced the overall results, as most participants from the study group were actively taking medication as a part of their treatment process around the time of measurement. Here, we investigated the association with olanzapine equivalent doses and found that only *m*/*z* 61, *m*/*z* 71, *m*/*z* 73, and *m*/*z* 79 were significantly associated with dose equivalents, but that the VOCs showing decreased concentrations in schizophrenia were not associated with antipsychotic dose. Additionally, the lack of change between the 14 days of follow-up did not make medication effects very likely. Nevertheless, it is possible that some of the identified VOCs were influenced from metabolising the administered medication at the time and, thus, these aspects need to be further investigated.

As another limitation of the investigations, it must be mentioned that only small patient collectives were examined. Thus, no generalisation of the results can be made at first. In addition, the medication effect must be further investigated since the results of individual masses showed significant differences. The effects from smoking must be excluded as much as possible to minimize falsification of the data.

In addition, it must be noted that patients were included in the study who collected the respiratory samples at home independently and without supervision, according to prior detailed instructions. In this, there is a certain residual risk of possible incorrect sample collection or undetected influencing factors on the breath sample, and so, this can be considered as a further limitation of the study. Otherwise, only stabilised patients without major limitations were included, of whom it can be assumed that sampling was performed correctly after previous training.

*m*/*z* 67 showed a relatively large standard deviation in the calculations, which should be taken into account in the evaluation of the detected differences and needs further investigation to consolidate the results.

In previous studies, the Tedlar bags were examined for possible contamination of VOCs. The study Trabue et al. showed, for example, a background contamination of the Tedlar bags with phenols (*m*/*z* 95), N-Dimethylacetamide (DMAC), *m*/*z* 88, or acetic acid (*m*/*z* 61) [24]. Beauchamp et al. and Ghimenti et al. could partially confirm these results and detected a contamination of the Tedlar bags with phenols and DMAC. [25,26] In our results, *m*/*z* 95 showed significant temporal changes with decreasing concentration during awakening and *m*/*z* 61 showed a significant relationship to the respective olanzapine equivalents. Accordingly, it must be said that an influence of the Tedlar bags of the results of *m*/*z* 61 and 95 cannot completely excluded and should be considered in further studies. For all participants, brand new and never-before-used Tedlar bags were used. Therefore, there were no residual VOCs in the Tedlar bags from other participants.

Breath gas analysis is a simple to perform and non-invasive analytical instrument that patients can perform partially on their own; accordingly, there was clinical applicability. Certainly, further studies are needed to confirm the results and to verify the accuracy and reproducibility and whether an establishment of a respiratory biomarker into the clinical routine can succeed.

## 3. Materials and Methods

### 3.1. Participants

In a study at Magdeburg University Hospital, the breath of a total of 22 patients with schizophrenia disorder was examined for VOCs in breath. The measurements were repeated after two weeks at three different time points, the first time immediately after waking up in the morning, after 30 min, and after 60 min. Moreover, 22 healthy controls were further investigated for comparison.

The participants were included following inclusion and exclusion criteria:

Patients had to have acute schizophrenia according to DSM-V, whether first episode or re-exacerbation, which was not drug-induced and, due to which, an inpatient admission occurred. Inclusion in the study and measurements were made only after sufficient clinical stabilisation.

Patients unable or unwilling to consent were excluded, as were patients with a history of severe hormonal dysfunction or severe neurological or internal diseases (e.g., multiple sclerosis, Parkinson’s disease, manifest hyper- or hypothyroidism, diabetes mellitus, major infections, or electrolyte imbalance). Similarly, participants with psychiatric comorbidities and acutely suicidal patients were excluded.

The study was approved by the local ethics committee and conducted in accordance with the Declaration of Helsinki. All participants received a written form detailing the study procedure and verbal explanations.

### 3.2. Breath Analysis

Breath gas analysis was performed by PTR-MS. Measurements were taken a total of three times on two measurement dates (baseline, 14-day follow-up); first, immediately after awakening, then, after 30 min, and, finally, after 60 min. The time points and time period were set to investigate the stability of VOCs over time and to capture physiological dynamic changes during the waking response. Patients were allowed to deliver the breath samples independently after detailed explanation and written instructions. Breath was collected in three Tedlar bags using special tubing. Prior to the measurements, the patients were fasting except for clear water, they were not allowed to smoke or brush their teeth and refrained from any physical activity.

At each time point, there was a two-minute rest period followed by a two-minute period with a certain predetermined breathing rhythm. After this, the sampling began. For this, the patients took one deep breath, opened the tube of the bag with their mouth by pushing it in, breathed deeply into the bag to fill it, and closed the bag again with their teeth. This manoeuvre was repeated at the above times.

Breathomics profiling was performed with high-sensitivity quadrupole PTR-MS instruments from IONICON Analytic GmbH, Innsbruck, Austria. In addition, mass calibration and compound identification as well as isotope correction were performed with PTR-MS-TOF using the interface of the PTR-MS Viewer software (version 3.3, IONICON Analytic GmbH, Innsbruck, Austria).

We measured the samples without further preparation via the PTR-MS within 5 h. According to a study from Beauchamp et al., VOCs can be preserved relatively stable in Tedlar bags for the first 10 h [25]. The PTR-MS measures the VOCs by taking in the breath gas through a drift tube and creating a reaction with protons, most commonly H_3_O^+^. Using a quadrupole-based analyser unit, we measured masses between *m*/*z* 19 and 200 [27]. In order to better classify masses, we made parallel investigations with PTR-MS and PTR-TOF-MS, as PTR-TOF-MS allowed a 100-fold more exact detection of masses with up to two decimal places. Masses selected for the current study and their tentative origins are indicated in Supplementary Table S3. PTR-TOF, however, did not allow the detection of the substrate, as it gave the masses in *m*/*z* and not a specific spectrum, where the substrate can be identified.

In a study by Sukul [28], VOCs in physiological respiratory hemodynamic and exhaled particles were investigated and a set of VOCs usually occurring in breath was described. Based on this and our own previous study findings [16,29], VOCs found in respiratory physiology were selected for investigation.

### 3.3. Clinical Investigations

Upon inclusion, general sociodemographic information such as age, sex, level of education, and employment status were collected. Moreover, medical history, prior medical records, family history as well as current and previous medication were also obtained.

All participants then had to undergo an extensive psychiatric evaluation. The patients’ status was investigated by using several clinical assessments: schizophrenic patients were evaluated by the positive and negative syndrome scale (PANSS) [30]. Furthermore, symptom severity as well as treatment response and efficacy were documented through the clinical global impression scale (CGI) [31].

In addition to that, all participants, including healthy controls, were given standardized self-assessment questionnaires to determine general psychopathology. Those included Beck’s Depression Inventory, Beck’s Anxiety Inventory (BAI) [32], and the (STAI I-II) [33]. Occupational and interpersonal adjustment were evaluated through the work and social adjustment scale (WSAS) [34], early infantile stressors were registered with the childhood trauma questionnaire (CTQ) [35], stressors during adolescence and, later in life, were assessed through the perceived stress scale (PSS) [36] and life event questionnaire (LEQ) [37,38]. Furthermore, the personality questionnaire NEO-Five Factor Inventory (NEO-FFI) was used and the Pittsburgh sleep quality index (PSQI) [39] was used to evaluate sleep quality of the participants. To record possible medication side effects, the frequency, intensity, and burden of side effects rating (FIBSER) [40] was used (Supplemental text).

### 3.4. Data Analysis

Statistical analysis was performed using SPSS (Version 28) and the GraphPad Prism7 program. The analysis was performed using bootstrap mixed model analysis. Bootstrap *p* values were adjusted for false discovery rate (FDR), *p* < 0.05 according to multiple testing. In bootstrap mixed model analysis, masses of VOCs formed the dependent variable. Age, smoking history, and BMI were used as between- participants covariates. In addition, the interactive effect between waking and time course was also considered.

In addition, we tested whether medication use influenced breathing by examining Pearson correlations between mass concentration and olanzapine equivalent dose [41]. The evolution of psychopathology over two weeks was analysed using *t*-test. For this purpose, the scores obtained in the PANSS (total PANSS, PANSS positive, PANSS negative) and BDI-II on the first test day were compared with the scores on the second test day (two weeks later). The original dataset was tested with Kolmogorov–Smirnov, D’Agostino and Pearson omnibus, and Shapiro–Wilk normality tests and they showed a normal distribution or a logarhythmic normal distribution, so that we could subsequently apply the paired *t*-test.”

The mean values of the collected scores of all participants on the first and on the second test day were calculated and correlated psychopathology with VOC concentrations using Spearman correlation. Intraclass correlation was also used to examine test–retest stability.

## 4. Conclusions

In conclusion, the analysis of breath for VOCs could be an interesting and promising approach for the development of a biomarker for schizophrenia. In particular, trimethylamine might be a potential signature for decision support and identifying a subgroup of patients responding to a certain antipsychotic group. In the future, the development of a biomarker could potentially have an impact on the early detection of the disease, treatment and, thus, patient outcome.

## Figures and Tables

**Table 1:** Demographic and clinical data for patients with schizophrenia (baseline and follow-up) and for healthy controls.

	Baseline SZ	Follow-Up SZ	*p* (Comparison between Baseline and Follow-Up)	Healthy Subjects	*p* (Comparison between Patients and Healthy Subjects)
PANSS *	51.20 ± 11.18	49.9 ± 10.75 2 missing pieces of data	*p* = 0.014		
PANSS positive *	11.50 ± 3.46	10.75 ± 2.92 2 missing pieces of data	*p* = 0.005		
PANSS negative *	13.05 ± 3.49	13.05 ± 3.44 2 missing pieces data	*p* = 0.500		
BDI-II *	20.73 ± 13.83	16.86 ± 13.02	*p* = 0.000384	1.91 ± 3.78	*p* = 3.1488 × 10^−16^
Medication group 1/2/3/4	7/5/2/7 one missing data	7/5/2/7 one missing piece of data			

Legend: * Data are presented as means ± standard deviation, SZ = schizophrenia patients, PANSS = positive and negative syndrome scale, BDI-II = Beck Depression Inventory, Medication group: 1 = risperidone, 2 = clozapine, olanzapine, quetiapine, 3 = aripiprazole, cariprazine, 4 = combination therapy.

**Table 2:** Mixed Model Analyses in patients and controls. Indicated are the mass concentration presented in means in ppb.

		*HC*		*p*		*SZ*	
	After Awakening	After 30 min	After 60 min		After Awakening	After 30 min	After 60 min
*m/z* 19 ↓	384,535.21 ± 159,212.53	411,666.15 ± 116,909.9	434,064.31 ± 114,846.25	*p*_1_ = 0.004 *p*_FDR_ = 0.011 *p*_2_ = 0.093 *p*_3_ = 0.896	300,524.02 ± 147,925.98	346,813.21 ± 134,503.77	375,629.56 ± 120,839.35
*m/z* 32	13,733.43 ± 31,038.39	4549.32 ± 7067.26	3524.65 ± 1945.92	*p*_1_ = 0.974 *p*_2_ = 0.032 *p*_3_ = 0.839	11,342.26 ± 22,900.95	6226.55 ± 8595.74	3952.63 ± 4174.1
*m/z* 33 ↓	516.51 ± 256.22	589.04 ± 252.09	615.64 ± 311.97	*p*_1_ = 0.000002 *p*_FDR_ = 0.000025 *p*_2_ = 0.288 *p*_3_ = 0.935	316.09 ± 256.92	349.94 ± 192.1	387.64 ± 247.07
*m/z* 35	2.03 ± 2.6	7705.57 ± 36,134.6	2.59 ± 3.27	*p*_1_ = 0.319 *p*_2_ = 0.371 *p*_3_ = 0.371	1.91 ± 2.99	1.09 ± 1.72	1.09 ± 1.19
*m/z* 42 ↑	30.32 ± 39.52	25.15 ± 21.87	20.64 ± 25.01	*p*_1_ = 0.000000004 *p*_FDR_ = 0.0000001 *p*_2_ = 0.650 *p*_3_ = 0.434	125.55 ± 109.1	167.30 ± 178.09	180.00 ± 200.99
*m/z* 45	239.57 ± 375.94	131.37 ± 75.08	98.70 ± 35.66	*p*_1_ = 0.913 *p*_2_ = 0.007 *p*_3_ = 0.877	212.91 ± 229.99	135.30 ± 66.25	110.73 ± 58.17
*m/z* 47	132.47 ± 83.38	830.64 ± 3397.48	322.14 ± 1060.86	*p*_1_ = 0.329 *p*_2_ = 0.561 *p*_3_ = 0.464	240.64 ± 531.82	163.32 ± 242.74	127.00 ± 105.89
*m/z* 57	31.39 ± 29.78	39.31 ± 32.43	30.98 ± 49.20	*p*_1_ = 0.138 *p*_2_ = 0.642 *p*_3_ = 0.678	50.64 ± 57.47	44.58 ± 41.51	38.00 ± 20.56
*m/z* 59 ↓	2265.93 ± 2180.02	2173.68 ± 1637.89	2344.93 ± 2220.62	*p*_1_ = 0.0009 *p*_FDR_ = 0.0028 *p*_2_ = 0.914 *p*_3_ = 0.908	1105.18 ± 1233.20	1330.55 ± 1336.75	1333.00 ± 1274.98
*m/z* 60 ↓	182.49 ± 165.02	132.39 ± 63.95	130.09 ± 80.03	*p*_1_ = 0.000006 *p*_FDR_ = 0.00005 *p*_2_ = 0.277 *p*_3_ = 0.273	77.18 ± 50.87	77.75 ± 43.07	77.00 ± 47.15
*m/z* 61	289.09 ± 524.24	133.16 ± 50.81	171.50 ± 380.04	*p*_1_ = 0.143 *p*_2_ = 0.246 *p*_3_ = 0.835	432.54 ± 800.32	309.62 ± 514.23	227.09 ± 318.22
*m/z* 63	36.94 ± 16.38	36.88 ± 21.44	31.77 ± 18.93	*p*_1_ = 0. 0.641 *p*_2_ = 0.753 *p*_3_ = 0.548	29.82 ± 24.54	35.77 ± 25.51	34.73 ± 21.66
*m/z* 67	38.22 ± 91.55	11.03 ± 17.58	7.48 ± 6.49	*p*_1_ = 0.618 *p*_2_ = 0.013 *p*_3_ = 0.795	27.36 ± 48.46	12.25 ± 16.19	5.73 ± 6.03
*m/z* 69 ↓	370.08 ± 260.74	299.69 ± 189.77	213.77 ± 127.33	*p*_1_ = 0.00023 *p*_FDR_ = 0.00096 *p*_2_ = 0.022 *p*_3_ = 0.264	196.00 ± 138.33	200.42 ± 139.57	155.27 ± 97.86
*m/z* 71	11.47 ± 6.21	8.91 ± 4.58	8.86 ± 6.85	*p*_1_ = 0.086 *p*_2_ = 0.449 *p*_3_ = 0.548	37.73 ± 101.83	31.73 ± 91.44	10.73 ± 13.23
*m/z* 73	13.98 ± 16.9	10.66 ± 7.42	10.57 ± 5.79	*p*_1_ = 0.022 *p*_2_ = 0.255 *p*_3_ = 0.776	27.00 ± 45.23	18.62 ± 23.48	17.00 ± 10.58
*m/z* 74 ↑	30.87 ± 28.43	28.10 ± 17.04	29.20 ± 12.20	*p*_1_ = 0.000018 *p*_FDR_ = 0.00011 *p*_2_ = 0.906 *p*_3_ = 0.901	17.27 ± 8.41	17.47 ± 12.12	16.27 ± 8.15
*m/z* 79	5.09 ± 7.55	2.57 ± 2.45	3.14 ± 5.79	*p*_1_ = 0.013 *p*_2_ = 0.518 *p*_3_ = 0.721	10.36 ± 21.07	10.00 ± 16.41	6.36 ± 8.66
*m/z* 87	126.77 ± 449.60	25.11 ± 21.01	17.91 ± 11.39	*p*_1_ = 0.483 *p*_2_ = 0.265 *p*_3_ = 0.392	38.91 ± 84.10	38.08 ± 80.26	22.55 ± 52.17
*m/z* 89 ↓	290.55 ± 293.63	312.16 ± 286.39	287.77 ± 267.26	*p*_1_ = 0.000033 *p*_FDR_ = 0.00017 *p*_2_ = 0.612 *p*_3_ = 0.897	98.09 ± 92.99	163.11 ± 277.28	102.27 ± 62.65
*m/z* 91	5.47 ± 4.32	6.13 ± 4.37	6.09 ± 5.47	*p*_1_ = 0.029 *p*_2_ = 0.405 *p*_3_ = 0.873	3.36 ± 2.92	4.91 ± 3.64	4.64 ± 3.67
*m/z* 93 ↑	2.57 ± 2.34	0.83 ± 1.60	3.05 ± 2.42	*p*_1_ = 0.00081 *p*_FDR_ = 0.0028 *p*_2_ = 0.083 *p*_3_ = 0.182	6.64 ± 8.20	4.09 ± 4.60	3.73 ± 4.20
*m/z* 95	708.63 ± 414.87	890.45 ± 327.40	854.39 ± 348.29	*p*_1_ = 0.197 *p*_2_ = 0.068 *p*_3_ = 0.376	586.64 ± 466.01	792.60 ± 467.37	896.18 ± 425.54
*m/z* 101	3.60 ± 3.66	3.32 ± 4.53	2.59 ± 2.39	*p*_1_ = 0.080 *p*_2_ = 0.833 *p*_3_ = 0.630	2.18 ± 3.20	2.00 ± 2.05	2.36 ± 2.80

Legend: ↑ = higher concentration in schizophrenia patients, ↓ = lower concentration in schizophrenia patients, _FDR_ = false discovery rate, *HC* = Health control, *SZ* = Schizophrenic patients, *p*_1_ = diagnosis, *p*_2_ = awakening, *p*_3_ = diagnosis * awakening, ± = standard deviation.

**Table 3:** Test–retest reliability as measured by intra class correlations for VOCs of baseline and follow-up investigations. Shown are results for all masses tested.

Correlation Coefficient in Classes				
	Correlation within the Class	95%-Confidence Interval		F-Test with True Value 0
		Lower Limit	Upper Limit	Sig.
*m*/*z* 32	0.098	−1.171	0.626	0.407
*m*/*z* 33	0.765	0.434	0.902	0.00081
*m*/*z* 35	0.708	0.298	0.879	0.00336
*m*/*z* 42	0.894	0.744	0.956	0.000002
*m*/*z* 45	0.615	0.072	0.840	0.017
*m*/*z* 47	0.643	0.141	0.852	0.011
*m*/*z* 57	0.372	−0.512	0.739	0.147
*m*/*z* 59	0.781	0.473	0.909	0.00049
*m*/*z* 60	0.801	0.534	0.920	0.00020
*m*/*z* 61	0.556	−0.070	0.816	0.035
*m*/*z* 63	0.836	0.605	0.932	0.00006
*m*/*z* 67	0.243	−0.824	0.686	0.265
*m*/*z* 69	0.759	0.419	0.900	0.00097
*m*/*z* 71	0.553	−0.077	0.814	0.036
*m*/*z* 73	0.475	−0.264	0.782	0.074
*m*/*z* 74	0.346	−0.576	0.728	0.169
*m*/*z* 79	0.701	0.280	0.876	0.003935
*m*/*z* 87	0.550	−0.083	0.813	0.037
*m*/*z* 89	0.022	−0.1355	0.594	0.479
*m*/*z* 91	0.591	0.018	0.831	0.023
*m*/*z* 93	0.600	0.036	0.834	0.021
*m*/*z* 95	0.069	−1.243	0.613	0.436
*m*/*z* 101	0.226	−0.864	0.679	0.281

## Data Availability

Not applicable.

## Supplementary Materials

The following supporting information can be downloaded at: https://www.mdpi.com/article/10.3390/molecules28114385/s1, Figure S1: Dynamic change of concentration of mass *m/z* 95; Figure S2: Association between olanzapine equivalents and concentration of *m/z* 71; Figure S3: Association between olanzapine equivalents and concentration of *m/z* 73; Figure S4: Shown is the Pearson-Correlation between the average concentration of mass *m/z* 79 and the olanzapine equivalents. Table S1: Mixed Model Analyses in patients and controls; Table S2: Pearson-correlation between the mass *m/z* 33, 42, 59, 60: Table S3: Preselected masses.
