# Supplementary material for: Dynamic Stability of Volatile Organic Compounds in Respiratory Air in Schizophrenic Patients and Its Potential Predicting Efficacy of TAAR Agonists
Source: Molecules. 2023 May 27;28(11):4385. doi: 10.3390/molecules28114385 (PMC10254278; doi:10.3390/molecules28114385)

Supplementary Table S1. Mixed Model Analyses in patients and controls. Indicated are the mass concentration presented in means in ppb

	HC			*SZ*		
	**Measurement 1**	**Measurement 2**	**Measurement 3**	**Measurement 1**	**Measurement 2**	**Measurement 3**
m/z 32	13733.43	4549.32	3524.65	11342.26	6226.55	3952.63
m/z 33 ↓	62.82	38.98	20.84	54.64	30.60	22.36
m/z 35	2.03	7705.57	2.59	1.91	1.09	1.09
m/z 42 ↑	30.32	25.15	20.64	125.55	167.30	178.00
m/z 45	239.57	131.37	98.70	212.91	135.30	110.73
m/z 47	132.47	830.64	322.14	240.64	163.32	127.00
m/z 57	31.39	39.31	30.98	50.64	44.58	38.00
m/z 59 ↓	2265.93	2173.68	2344.93	1105.18	1330.55	1333.00
m/z 60 ↓	182.49	132.39	130.09	77.18	77.75	77.00
m/z 61	289.09	133.16	171.50	432.54	309.62	227.09
m/z 63	36.94	36.88	31.77	29.82	35.77	34.73
m/z 67	38.22	11.03	7.48	27.36	12.25	5.73
m/z 69 ↓	370.08	299.69	213.77	196.00	200.42	155.27
m/z 71	11.47	8.91	8.86	37.73	31.73	10.73
m/z 73	13.98	10.66	10.57	27.00	18.62	17.00
m/z 74 ↑	30.87	28.10	29.20	17.27	17.47	16.27
m/z 79	5.09	2.57	3.14	10.36	10.00	6.36
m/z 87	126.77	25.11	17.91	38.91	38.08	22.55
m/z 89 ↓	290.55	312.16	287.77	98.09	163.11	102.27
m/z 91	5.47	6.13	6.09	3.36	4.91	4.64
m/z 93 ↑	2.57	0.83	3.05	6.64	4.09	3.73
m/z 95	708.63	890.45	854.39	586.64	792.60	896.18
m/z 101	3.60	3.32	2.59	2.18	2.00	2.36

Legend: HC= Health control, SZ= Schizophrenic patients

Supplementary Table S2: Shown is the Pearson-correlation with significance level p between the mass m/z 33, 42, 59, 60

		m/z 33	m/z 42	m/z 59	m/z 60	m/z 69	m/z 74	m/z 89	m/z 93
m/z 33	Pearson-correlation	1	0.334	0.135	0.118	0.378	0.243	0.388	0.354
	p		0.000089	0.122	0.176	0.000008	0.005	0.000004	0.000032
m/z 42	Pearson-correlation	0.334	1	0.090	0.055	0.322	0.041	0.153	0.392
	p	0.000089		0.307	0.535	0.000162	0.639	0.080	0.000003
m/z 59	Pearson-correlation	0.135	0.090	1	0.784	0.028	0.014	0.011	0.168
	p	0.122	0.307		$1.0568E^{-28}$	0.754	0.876	0.897	0.054
m/z 60	Pearson-correlation	0.118	0.055	0.784	1	0.021	0.349	0.172	0.078
	p	0.176	0.535	$1.0568E^{-28}$		0.809	0.000041	0.049	0.371
m/z 69	Pearson-correlation	0.378	0.322	0.028	0.021	1	0.282	0.365	0.267
	p	0.000008	0.000162	0.754	0.809		0.001	0.000017	0.002
m/z 74	Pearson-correlation	0.243	0.041	0.014	0.349	0.282	1	0.588	0.060
	P	0.005	0.639	0.876	0.000041	0.001		$1.1929E^{-13}$	0.493
m/z 89	Pearson-correlation	0.388	0.153	0.011	0.172	0.365	0.588	1	0.194
	P	0.000004	0.080	0.897	0.049	0.000017	$1.1929E^{-13}$		0.026
m/z 93	Pearson-correlation	0.354	0.392	0.168	0.078	0.267	0.060	0.194	1
	p	0.000032	0.000003	0.054	0.371	0.002	0.493	0.026	

T,

Supplementary Table S3 Preselected VOCs for analysis

NH_3	Ammonia	18
CH_2CO	Ketene	31
O2	Oxygen	32
H_2S	Hydrogen Sulfide	35
H2O	Water	19
C_2H_3N	Acetonitrile	42
CO2	Carbon Dioxide	45
C2H4O	Acetaldehyde	45
CH_2O_2	Formic Acid	47
C2H5OH	Ethanol	47
C3H4O	Acrolein	57
$(CH_3)_2CO$	Acetone	59
N(CH3)3	Trimethylamine	60
CH_3COOH	Acetic Acid	61
C3H8O	Isopropanol	61
CH_3CH_2SH	Ethanethiol	63
C_5H_6	Cyclopentadien	67
C4H4O	Furan	69
C5H8	Isoprene	69

C4H6O	Crotonaldehyde	71
C5H10	Penten	71
C4H8O	Butanone	73
$CH_3(CH_2)_3NH_2$	N-Butylamine	74
C6H6	Benzol	79
C4H6O2	y-Butyrolactone	87
C5H10O	2- Pentanone	87
C4H8S	Tetrahydrothiophen	89
C4H8O2	Ethylacetate	89
$CH_3CH_2CH_2CO_2H$	Butyric Acid	90
C4H10S	2- Butanethiol	91
C7H8	Toluol	93
C6H6O	Phenol	95
C5H8O2	Acetylacetone	101
C6H12O	Hexanal	101
C10H16	Adamantan	137

Supplementary Figure S1. Dynamic change of concentration of mass m/z 95 Shown is the average concentration of mass m/z 95 at the three different measurement time points over a 14-day time course. A significant awakening effect was detected.

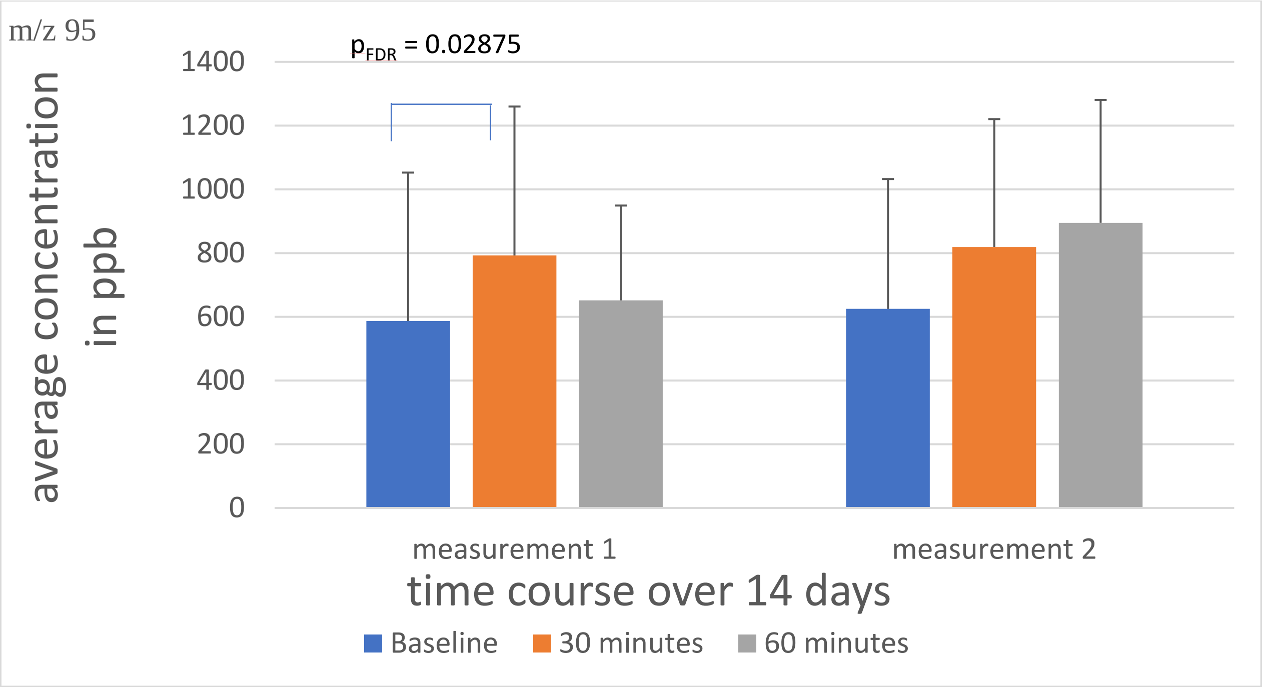

Supplementary Figure S2. Association between olanzapine equivalents and concentration of m/z 71 Shown is the Pearson-Correlation between the average concentration of mass m/z 71 and the olanzapine equivalents.

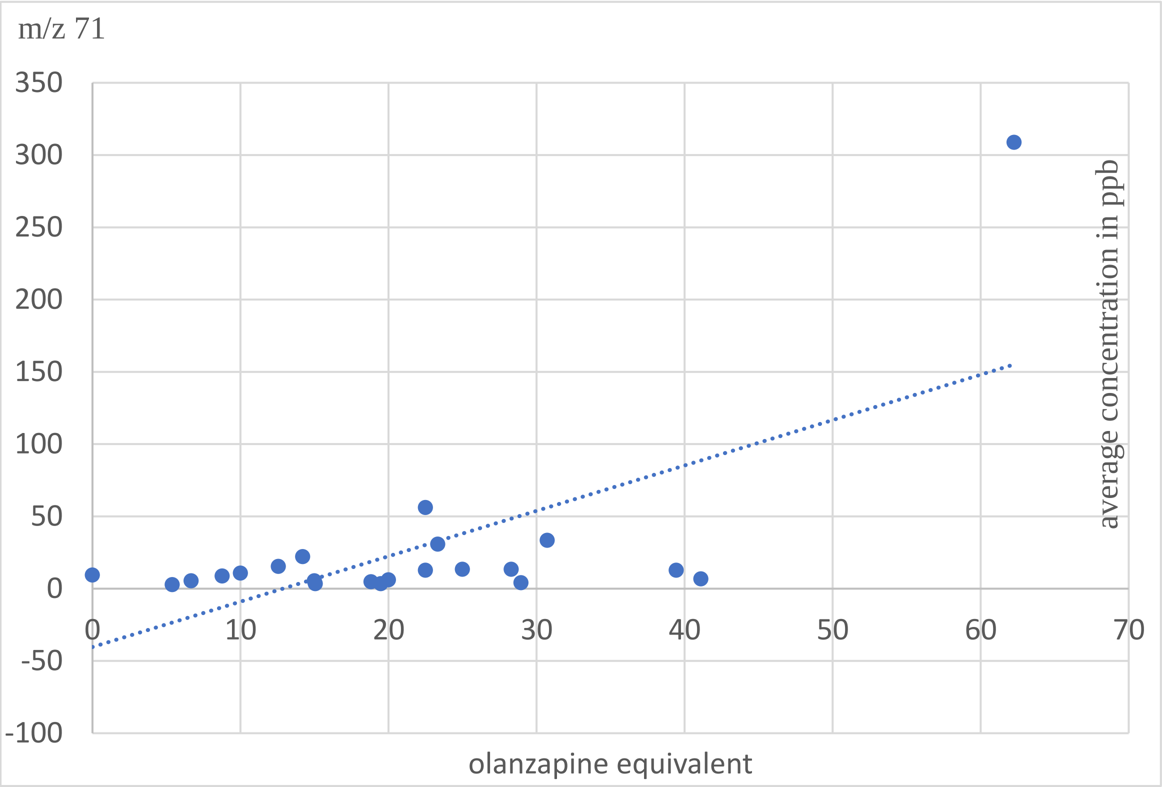

Supplementary Figure S3. Association between olanzapine equivalents and concentration of m/z 73 Shown is the Pearson-Correlation between the average concentration of mass m/z 73 and the olanzapine equivalents.

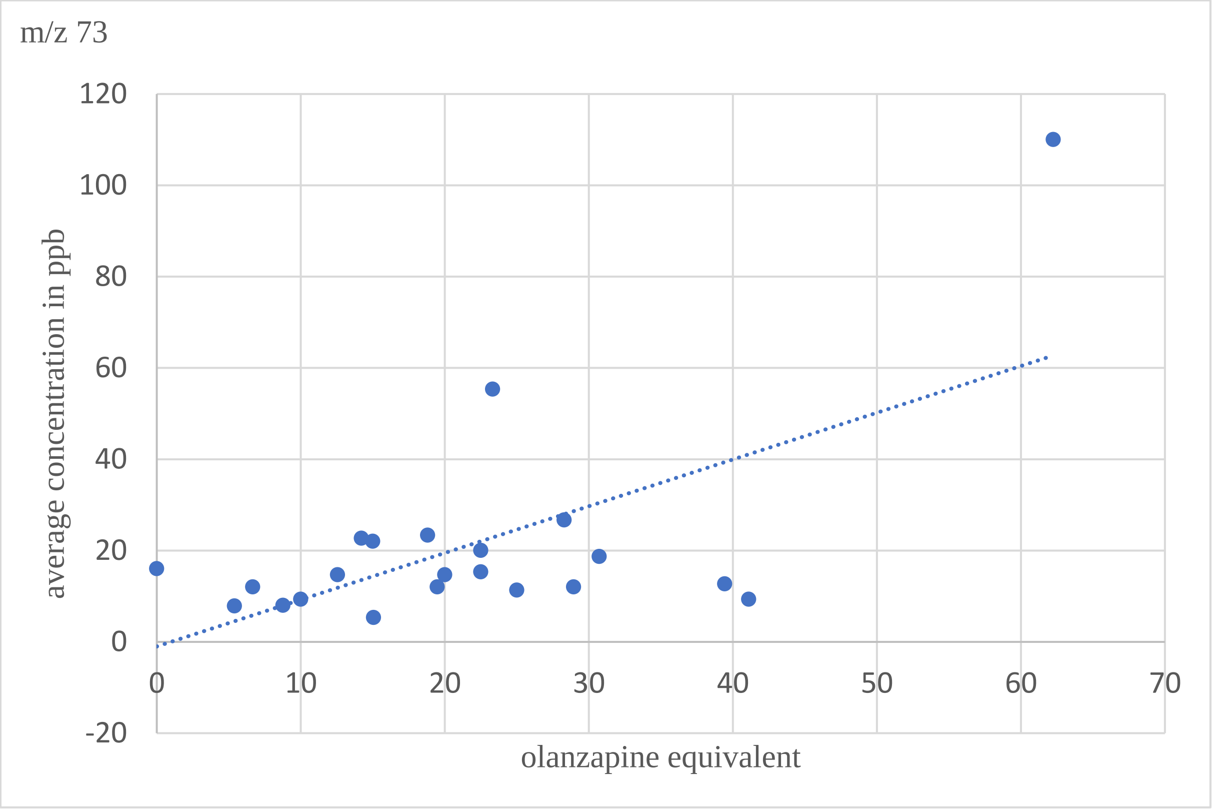

Supplemental Figure 4. Shown is the Pearson-Correlation between the average concentration of mass m/z 79 and the olanzapine equivalents.

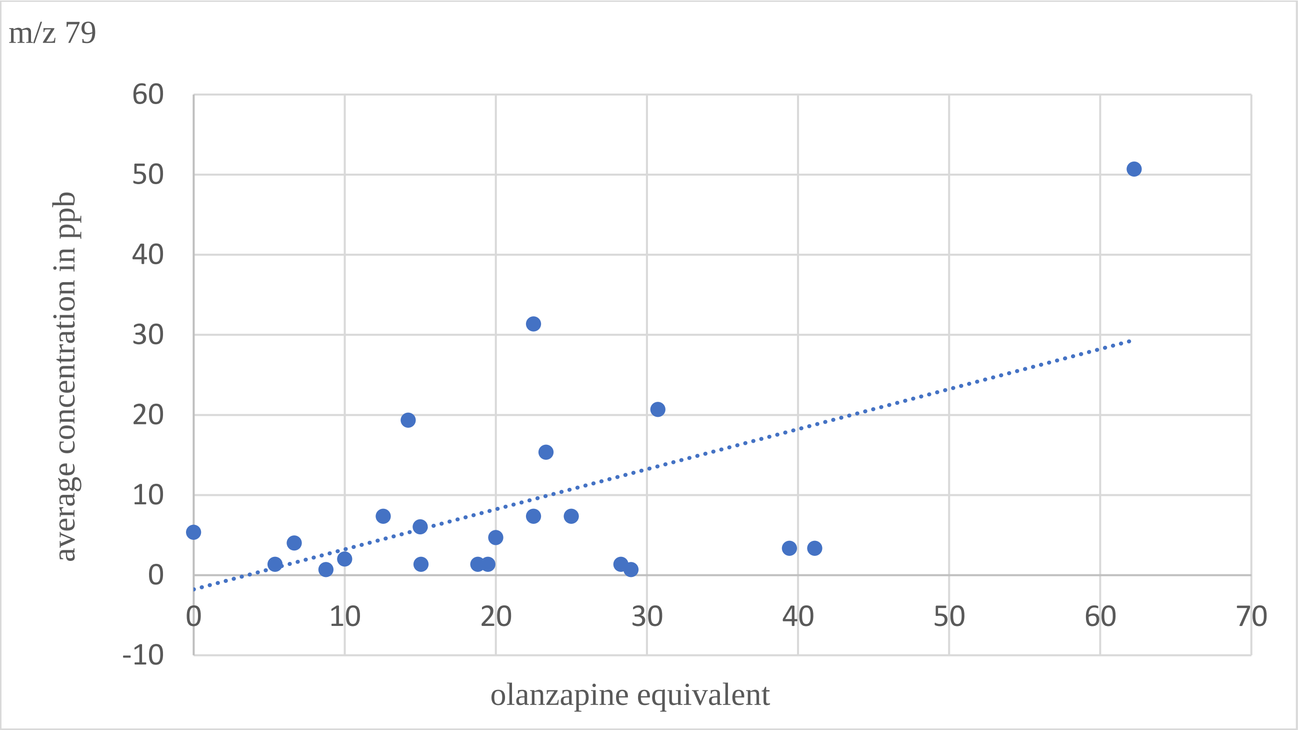

Supplement: Supplementary file 1 [file molecules-28-04385-s001.zip › molecules-2398639-supplementary.pdf]
